# Biological invasions, climate change and genomics

**DOI:** 10.1111/eva.12234

**Published:** 2014-12-09

**Authors:** Steven L Chown, Kathryn A Hodgins, Philippa C Griffin, John G Oakeshott, Margaret Byrne, Ary A Hoffmann

**Affiliations:** 1 School of Biological Sciences, Monash UniversityClayton, Vic., Australia; 2 Department of Genetics, Bio21 Institute, The University of MelbourneParkville, Vic., Australia; 3 CSIRO Land and Water Flagship, Black Mountain LaboratoriesCanberra, ACT, Australia; 4 Science and Conservation Division, Department of Parks and Wildlife, Bentley Delivery CentreBentley, WA, Australia; 5 Departments of Zoology and Genetics, Bio21 Institute, The University of MelbourneParkville, Vic., Australia

**Keywords:** adaptation, admixture, climate change, decision framework, genomics, hybridization, invasive species, management

## Abstract

The rate of biological invasions is expected to increase as the effects of climate change on biological communities become widespread. Climate change enhances habitat disturbance which facilitates the establishment of invasive species, which in turn provides opportunities for hybridization and introgression. These effects influence local biodiversity that can be tracked through genetic and genomic approaches. Metabarcoding and metagenomic approaches provide a way of monitoring some types of communities under climate change for the appearance of invasives. Introgression and hybridization can be followed by the analysis of entire genomes so that rapidly changing areas of the genome are identified and instances of genetic pollution monitored. Genomic markers enable accurate tracking of invasive species’ geographic origin well beyond what was previously possible. New genomic tools are promoting fresh insights into classic questions about invading organisms under climate change, such as the role of genetic variation, local adaptation and climate pre-adaptation in successful invasions. These tools are providing managers with often more effective means to identify potential threats, improve surveillance and assess impacts on communities. We provide a framework for the application of genomic techniques within a management context and also indicate some important limitations in what can be achieved.

## Introduction

Biological invasions constitute a major environmental change driver, affecting conservation, agriculture and human health. Invasive alien species (IAS) impacts include, alterations of ecosystem features such as hydrology, fire regimes, food webs, soil nutrients and nutrient cycling (Hänel and Chown 1998; Asner and Vitousek 2005; Van Wilgen 2009; Veldtman et al. 2011); negative effects on populations (Blackburn et al. 2004; Butchart et al. 2010; Ziska et al. 2011); facilitation of further invasion and its associated impacts (O'Dowd et al. 2003); changes to evolutionary trajectories, such as by hybridization (McDonald et al. 2008); and evolutionary shifts in species responding to the new introductions (Strauss et al. 2006). Much attention has therefore been given to understanding the invasion process. A broadly accepted perspective on biological invasions now exists that distinguishes the stages of invasion and the mechanisms that are involved in each stage (Blackburn et al. 2011).

Although the field has grown rapidly (Mooney and Hobbs 2000; Sax et al. 2005; Richardson 2011; McGeoch et al. 2012; Spear et al. 2013), several significant challenges remain. Foremost among these is limited understanding of the mechanisms underlying each of the key transitions in the invasion process, reflected by periodic calls for additional work in these areas (e.g. Puth and Post 2005; Hulme et al. 2008). Most recently, attention has turned to the quantification and forecasting of IAS impacts. Several reviews have concluded that current knowledge and capability in this area are inadequate, rendering management responses either ineffective or inefficient (Pyšek et al. 2012; Hulme et al. 2013; Ricciardi et al. 2013; Simberloff et al. 2013).

Further challenges are presented by the simultaneous increase in the impacts of other environmental change drivers. Biological invasions (e.g. Lambdon et al. 2008; Chown et al. 2012; Richardson and Ricciardi 2013) are not the only forms of change increasing in frequency and extent. Climate change, as a consequence of anthropogenic greenhouse gas emissions, is continuing, with indications that the rate of change may be increasing owing to a rise in CO_2_ emissions (Rignot et al. 2011). Similarly, landscapes continue to change through human interventions (Barnosky et al. 2012). Exploitation is ongoing with apparently little abatement, resulting in substantial population declines, even within protected areas (Chown 2010; Craigie et al. 2010; Jackson 2010; Laurance et al. 2012). While some forms of pollution have declined notably, others have come to replace these (Sutherland et al. 2010, 2012).

Although the form of the interactions between biological invasions and these other environmental change drivers has yet to be fully generalized (Darling and Côté 2008; Bradley et al. 2010; Treasure and Chown 2014), the current view is that interactions are likely most often to be synergistic (Brook et al. 2008; Walther et al. 2009). Moreover, the simultaneous effects of environmental change drivers on indigenous species, and human responses to counter at least some of the effects of environmental change, such as through managed relocation (Schwartz et al. 2012), are likely to make management decisions about biological invasions much more difficult than in the past (Webber and Scott 2012).

The field of invasion science has recognized many of these challenges and research directions are clearly changing to meet them (McGeoch et al. 2012; Ricciardi et al. 2013; Simberloff et al. 2013). Much of this new work remains ecological in nature (at the organismal, population, species or ecosystem levels), including work that aims to guide management interventions to address the impacts of biological invasions (Richardson and Pyšek 2012; Blackburn et al. 2014). Nonetheless, from early on in the development of the field, several other approaches have been considered. Among these, the utility of genetics and the significance of evolutionary processes have long been recognized (e.g. Baker 1974). However, it is only recently that they have seen a resurgence of interest (Huey et al. 2000; Callaway and Maron 2006; Dormontt et al. 2011; Lawson Handley et al. 2011). Indeed, the significance of genetic approaches and an evolutionary perspective are now recognized as important not only for understanding the ability of species to progress along the stages of invasion (e.g. Pandit et al. 2011; Richardson and Pyšek 2012), but also for improving management interventions that might reduce the rates and impacts of invasions (Lawson Handley et al. 2011; Prentis and Pavasovic 2013). Examples include better genotypic matching of biocontrol agents and their hosts, and understanding the coevolutionary dynamics of invasive species and those with which they interact (Phillips and Shine 2006; McDonald et al. 2008; Gaskin et al. 2011). Nonetheless, additional scope exists for modern genetic approaches, and notably genomics, to contribute to the understanding and forecasting that is required to limit the rates and impacts of biological invasions, especially given the expectation of interactions with other global change drivers.

**Figure 1:**
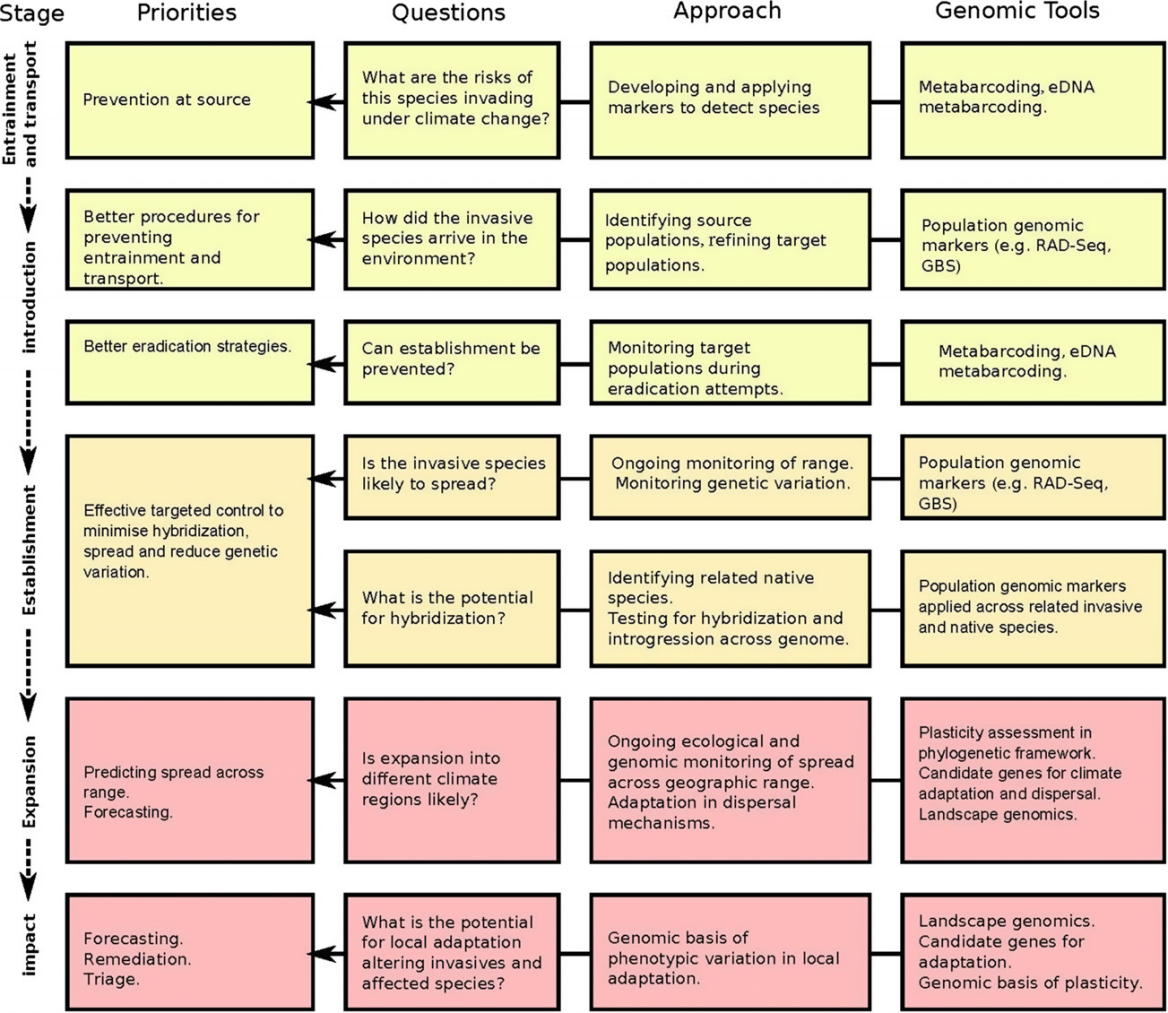
Genomic tools that can add to capacity for understanding risk and monitoring management actions at each stage of the invasion process. Tools can assist in the initial detection of an invasion, monitoring its spread after the initial detection and understanding the potential for invasions to expand and impact native species following evolutionary adaptation. Management priorities and questions relevant to each stage are also shown.

Here, we focus on the use of genomics to understand the changing form of invasion with climate change. We briefly review the contributions that genetic and evolutionary approaches have already made, discuss recent technological developments and then highlight further potential of this approach. We base our overview on the unified framework for biological invasions (Blackburn et al. 2011), recognizing that each of the stages in the invasion process can be mitigated by an accompanying management response (Fig.1). In addition, although various views exist about how invasive species should be defined (Pyšek et al. 2004; Valéry et al. 2008), here, to avoid uncertainty (McGeoch et al. 2012), we adopt the definition provided by Richardson et al. (2011). In consequence, we are concerned with the processes by which species are transported outside their natural range through direct or indirect anthropogenic intervention and become established, proliferate and interact with the biota in their new ranges.

## Genetic and evolutionary studies of invasive organisms

An increasing number of studies have documented evolutionary changes in invasive populations, typically over ecological timescales (Lee 2002; Prentis et al. 2008; Whitney and Gabler 2008). These studies have shown that evolutionary adaptation can be rapid in a broad range of invasive organisms. Examples include copepods adapting to different salinity levels (Lee and Petersen 2002), soapberry bugs adapting to new host plants (Carroll et al. 2001), and invasive weeds adapting through mimicry to evade eradication (Barrett 1983). Moreover, several of the documented examples of rapid evolutionary adaptation involve responses to climate change over a few decades. These include the evolution of *Drosophila* body size and inversion frequencies following invasion across continents with latitudinal climate variation (Huey et al. 2000), the evolution of rabbits along a heat gradient following invasion into Australia (Williams and Moore 1989), and changes in flowering time in *Solidago* goldenrods following introduction into Europe (Weber and Schmid 1998).

Recent work has continued to reinforce the notion that evolutionary genetic changes in invasive species can be rapid and often involve responses to climate. For instance, purple loosestrife, *Lythrum salicaria,* has invaded wetlands across an introduced range that covers a latitudinal gradient extending in eastern North America from 39°N to 48.5°N. Along this gradient, Colautti and Barrett (2013) showed an adaptive cline in flowering time, with earlier flowering at the northern invasion front, where it increases survival and fruit set, but with selection against earlier flowering in the south where it decreases vegetative growth. Climate adaptation involving changes in flowering traits has been recorded in several other invasive plant species (Moran and Alexander 2014). Evolution of traits in response to climate in invasive species has also been found for a range of animals. In *Drosophila subobscura*, introduced into Chile in the late 1970s, evolutionary divergence in thermal preferences among populations from different climates has weakened clines in chromosomal inversion (Castañeda et al. 2013). Likewise, physiological tolerances appear to have diverged in response to climate in the mite *Halotydeus destructor* (Hill et al. 2013). Several researchers have argued that evolution in invasive species can provide general answers to questions of whether species will be able to adapt to rapid climate change (Moran and Alexander 2014), including those about the speed of evolutionary change, the specific nature of the change that has occurred and factors that might limit evolutionary change.

Apart from their use to investigate adaptive changes, genetic tools have also been used to understand the dynamic nature of invasive processes. In particular, genetic markers have been widely used to detect invasive organisms, investigate patterns of historical movement in invasive species and examine the role of genetic variation in ensuring the success of invasions (Armstrong and Ball 2005; Lawson Handley et al. 2011; Blanchet 2012). Most past studies have focussed on markers thought to be selectively neutral, initially allozymes and then later microsatellites, AFLPs and other DNA markers. These markers can indicate the status of invasive species, origin of invasive populations and the levels of genetic variation in invading populations when compared to the populations of origin. Recent examples include tracking invasions of the winter annual *Geranium carolinianum* in China from multiple origins (Shirk et al. 2014), identifying a key translocation event in the invasion by a coregonid fish of a major Scandinavian watershed (Præbel et al. 2013), the use of mtDNA markers and microsatellites to trace the invasion history of eastern mosquitofish (*Gambusia holbrooki*) into Australia (Ayres et al. 2012) and determining multiple origins of invasive acacias in South Africa and South Australia (Le Roux et al. 2011; Millar et al. 2012). Genetic markers have also provided much information on the biology of invasive species, such as modes of reproduction (Weeks et al. 1995; Ali et al. 2014; Molins et al. 2014), movement patterns and rates (Bronnenhuber et al. 2011; Kirk et al. 2011; Berthouly-Salazar et al. 2013) and predator–prey relationships (Kasper et al. 2004; Blanchet 2012; Valdez-Moreno et al. 2012).

These types of studies are now being supplemented with a range of genomic and transcriptomic approaches that are providing answers to questions about the origins of invasive populations at a new level of resolution, providing new ways of monitoring for invasive organisms, and leading to an understanding of the nature of the changes that underlie adaptive shifts. We consider the promise provided by these studies within the context of detecting and managing biological invasions as climate change proceeds, and within a framework of the different steps involved in detecting and responding to an invader, understanding its source, and then tracking its impact on the surroundings (Fig.1).

## Technology

### Model and nonmodel organisms

The genomes of several invasive species have now been sequenced and assembled as well as annotated to differing extents. These include the tunicate *Ciona intestinalis* (Dehal et al. 2002), dengue mosquito vector *Aedes aegypti* (Nene et al. 2007), argentine ant *Linepithema humile* (Smith et al. 2011), fire ant *Solenopsis invicta*, (Wurm et al. 2011), diamondback moth *Plutella xylostella* (You et al. 2013), domestic cat *Felis catus* (Pontius et al. 2007) and mallard *Anas platyrhynchos* (Huang et al. 2013). Then, there are the genomes of model species that are also invasive, which include *Drosophila* species such as *D. melanogaster* and *D. suzukii* (Adams et al. 2000; Chiu et al. 2013), rats and mice (Waterston et al. 2002; Gibbs et al. 2004), and some weedy plants, such as the castor bean (Chan et al. 2010). Some of these species are useful for climatic studies, based on evidence for geographic variation in climate responses in species such as the drosophilids (Hoffmann et al. 2003) and *C. intestinalis* (Dybern 1965).

Many genomic approaches can be applied to population genetic studies of nonmodel species that lack a reference genome assembly. The use of sequence tagging and ‘Pool-Seq’ technologies, together with relatively inexpensive RNA-Seq, genotyping-by-sequencing (GBS) and other reduced-representation library methods, means that data on many thousands of single nucleotide polymorphisms (SNPs) can be collected at moderate cost on multiple samples from a population without a reference genome (Box 1) (e.g. Hohenlohe et al. 2010; Elshire et al. 2011; Bi et al. 2013; Neves et al. 2013; Yeaman et al. 2014). However, even draft reference genomes or genomes from related species can be useful when implementing these approaches to identify variants. The expansion of reference genomes across a wider array of species will facilitate the study of invasion genomics. Several impediments to genome construction still exist (Ellegren 2014), although new technologies are emerging to overcome them and the cost of short-read sequencing is declining. Nonetheless, many plants and animals have large genomes, and the common occurrence of higher ploidy, especially in invasive plants, adds significant difficulty and expense. Higher ploidy also complicates other approaches such as SNP identification and analyses (see below).

**Box 1: Sequencing approaches for ecological genomics**

Whole-genome resequencing is cost-prohibitive and unnecessary in many cases. As an alternative, several methods of genome reduction make it possible for many individuals to be combined in one sequencing run while maintaining high coverage. This results in the reliable identification of SNPs and genotypes for each individual at a subset of regions throughout the genome.

Restriction-enzyme-based reduced-representation sequencing: Genomic DNA is digested with specific restriction enzymes, followed by ligation of adaptors, amplification and sequencing. Several methods employ this strategy, including genotyping-by-sequencing (GBS; Elshire et al. 2011) and RAD-Seq (Hohenlohe et al. 2010). Repetitive regions of genomes can be avoided using methylation-sensitive enzymes (Elshire et al. 2011). This approach is relatively inexpensive and easy to use in nonmodel organisms, but can suffer from biases such as those associated with the loss of restriction sites in some individuals (Gautier et al. 2013).

RNA-Seq: Sequencing mRNA from the genes that are expressed in the whole organism or specific sampled tissues at a particular point in time and development and under specific conditions (the ‘transcriptome’ of a whole organism or specific tissue; Wang et al. 2009). This method targets the expressed portion of the genome (protein-coding sequences and untranslated regions of genes). Gene expression and sequence information is simultaneously identified, as it is possible to compare counts of reads that map to genes and identify variants given sufficient expression of that gene in each individual.

Targeted sequence capture by hybridization: This approach uses oligoprobes to enrich specific regions of the genome for subsequent next-generation sequencing. The scale of the capture can range from a handful to several thousand targeted loci, making it appropriate for wide range of projects. However, prior genomic knowledge is essential for the development of the probes (e.g. through RAD-Seq or RNA-Seq projects). The development of noncommercial protocols and PCR-based probes (Peñalba et al. 2014) is making this approach more attractive for smaller-scale sequencing projects in nonmodel species. Several alternative methods are available that enrich target regions prior to sequencing (for review, see Mamanova et al. 2010).

Gene-space sequencing: Many genomes are replete with repetitive regions along with genes present in high copy number (e.g. chloroplast genes), and whole-genome sequencing without reducing their quantity can be a substantial waste of sequencing space. Consequently, some approaches seek to deplete these regions rather than enrich specific portions of the genome (e.g. Matvienko et al. 2013).

**Glossary of genetic terms**

**Table:** 

Genetic architecture:	the genetic underpinnings of phenotypic traits, including the number loci, their effect sizes and location in the genome.
Linkage disequilibrium:	the nonrandom association between alleles at two or more loci.
Quantitative trait locus (QTL):	a genomic area associated with variation in a quantitative trait in the progeny of a genetic cross.
Standing genetic variation:	existing variation in a population as opposed to variation that results from new mutations.
Wright fixation index (*F*_ST_):	the proportion of the total genetic variability that occurs among populations. It is a measure of the level of population genetic differentiation.

An important feature of invasive species for evolutionary studies is that the introduction and spread of the invader is often documented (Baker 1974), and in many cases, this includes historic collections. Genomic studies are benefiting from increased progress in obtaining information from specimens stored in herbariums, museum collections or recovered from natural repositories such as sediment layers in lake bottoms (Krehenwinkel and Tautz 2013; Martin et al. 2014). Such studies are particularly applicable to invasive organisms that are often represented well in collections with longitudinal sampling. These specimen records often cover periods over which populations have been exposed to relatively rapid climate change (such as between 1970 and 2000) (Hansen et al. 2013). More formal approaches are also now being applied to secure the preservation of material across time, such as the resurrection initiative for plants (Franks et al. 2008).

In future, methods can be employed that maximize the genomic information obtained from such limited material, rather than focusing on a few anonymous markers. Whole-genome resequencing has been used successfully for historic samples (Rowe et al. 2011), although contamination by nontarget DNA can make this expensive proposition even more cost-prohibitive. Sequence capture, which utilizes probes to enrich target sequences (e.g. Bi et al. 2013) (Box 1), offers a compromise between minimizing sequencing costs and maximizing sequence information from target regions of the study organism. Pooling individual samples together prior to either whole-genome (e.g. Futschik and Schlotterer 2010) or reduced-representation (e.g. Vandepitte et al. 2012) sequencing is another approach that can greatly reduce costs, while still providing useful information about the genomic extent and location of variation and divergence. Genome-wide analysis of historic DNA in invasive species will provide exciting opportunities to assess the temporal dynamics of evolutionary change during colonization and spread, and over periods of rapid climate change. It can also provide evidence of past changes in population size across time through coalescence methods (do Amaral et al. 2013) and indicate parts of the genome that have been affected by selection and population processes (Excoffier et al. 2009). Such data will improve inference of invasion history and aid in the identification of the genetic basis of recent adaptation.

### Top-down and bottom-up approaches

Ecological and landscape genomics are approaches that incorporate information about the phenotype, genotype and the local environment to make inferences about the loci involved in local adaptation. ‘Bottom-up’ approaches use the genomic signatures of selection to identify loci likely important for adaptation and do not rely on phenotypic information (Wright and Gaut 2005). Several different methods are used, such as tests examining the site frequency spectrum or the extent of linkage disequilibrium, which can indicate the location of a recent selective sweep (Smith and Haigh 2007; Thornton et al. 2007). The popular *F*_ST_ outlier method identifies regions of the genome under divergent selection by determining which sites have strong differentiation among populations relative to the entire genome or a set of putatively neutrally evolving loci (Lewontin and Krakauer 1973; Beaumont and Nichols 1996; Foll and Gaggiotti 2008; Steane et al. 2014). Another approach involves identifying associations between alleles and the local environment, while controlling for population structure, to make inferences about local adaptation (e.g. Coop et al. 2010; Günther and Coop 2013). Many of these tests of selection could be applied to invasive species to identify the genes associated with adaptation during invasion and in responses to climate change. These methods are appealing because genes under selection during invasion can be identified without *a priori* identification of specific functional traits and do not require common garden experiments. However, the loci identified may only be in linkage disequilibrium with the functional site(s). Moreover, population structure can hinder the ability of these tests to correctly identify adaptive loci, and certain demographic scenarios, particularly those involving nonequilibrium conditions, have been shown to generate a large number of false positives (Lotterhos and Whitlock 2014). A critical assessment of how complicated invasion histories, including founder events and genetic admixture, might impact the ability of these methods to detect loci under very recent divergent selection has not yet been conducted, but these processes could represent an impediment to the successful implementation of these tests in some invasive species.

‘Top-down’ methods offer a complementary approach for identifying the genetic basis of adaptation (Wright and Gaut 2005; Barrett and Hoekstra 2011). Here, the genetic basis of specific traits under selection during invasion can be dissected by looking for the cosegregation of these traits and genetic markers. Quantitative trait loci (QTL) mapping uses pedigreed populations descended from known parents. Linkage disequilibrium between genetic markers and causal loci enables the identification of genomic regions segregating with the phenotypes of interest. This method limits the identification of loci to those present in the initial cross, and the genomic resolution is low due to high linkage disequilibrium in the mapping population. Association mapping studies use unpedigreed mapping to look for associations between traits and markers, either using a candidate gene approach or genome-wide markers (GWAS studies) (Box 1). Such studies can be prone to false positives through spurious associations brought about by population structure, although there are statistical means to control for these effects (Tian et al. 2008). The approaches offer greater resolution than QTL studies due to the lower linkage disequilibrium found in natural populations relative to pedigreed mapping populations.

Top-down and bottom-up approaches each have their own advantages and disadvantages (Wright and Gaut 2005; Barrett and Hoekstra 2011; Le Corre and Kremer 2012; Sork et al. 2013), suggesting that tackling the problem from both ends will often yield the most useful results. However, there are biases common to all these methods that may obscure the genetic architecture of adaptation during invasion, or to climate change signals (Moran and Alexander 2014). For example, detecting small effect loci underlying polygenic traits has been a major challenge regardless of the method applied (Pritchard et al. 2010; Le Corre and Kremer 2012). Well-powered QTL and GWAS studies appear to be an effective solution to this problem (Bloom et al. 2013), and new analytical methods that attempt to integrate information from GWAS with allele frequency data from many populations are providing a powerful way to identify the signal of local adaptation in polygenic traits (Turchin et al. 2012; Berg and Coop 2014).

Although there have been few studies that have applied these approaches to identify genes important for adaptation during invasion, the growth of genomic data in nonmodel organisms suggest that these methods will soon be commonplace for invasive species. As strong selection will be required to see evolutionary changes over short time scales, theory suggests that large-effect loci (Yeaman and Whitlock 2011) or perhaps clusters of adaptive loci may be more likely to underlie a rapid response to selection. However, genetic changes from standing variation as opposed to new mutations are likely to be essential for adaptation during invasion, due to the waiting time required for new mutations (Barrett and Schluter 2008; Prentis et al. 2008). This suggests that polygenic adaptation from standing variation present in the original introduction is likely, so adaptation through multiple loci of small effect may also play an important role in the invasion. Both empirical and theoretical studies are needed to determine which features of the genetic architecture of rapid adaptation, if any, might distinguish it from longer-term adaptive evolution and if differences in genetic architecture could impact the propensity of organisms to become invasive and/or rapidly adapt to continuing climate change.

The identification of candidate ‘invasion’ genes using these top-down and bottom-up approaches is only the first step. Comparisons across species will be essential for determining whether there are similar functional groups, genes or genetic pathways that frequently evolve during invasion and in responses to changing environments further precipitated by local climate change. This might be expected, for example, if invading species commonly evolve along similar fitness trade-offs in response to changes in the biotic and abiotic environment in the introduced range (Blossey and Notzold 1995; Bossdorf et al. 2005; He et al. 2010). The context dependence and sometimes idiosyncratic nature of invasion and adaptation may, however, make such generalities unlikely (though see also Richardson and Pyšek 2006, 2012). Experimental studies, where possible, will be important to assess fitness effects of these candidate loci in nature, while controlling for genetic background (Barrett and Hoekstra 2011). The development of more model invasive species amenable to reverse genetics will be key for dissecting the functional role of candidate loci and will be a step forward for invasion genomics (Stewart et al. 2009).

## The initial stages – entrainment risk and transport

The introduction of alien species is either intentional, usually for economic benefit of some form, or unintentional, owing to the fact that species move around in all kinds of ways and include human vectors and human-mediated pathways in their dispersal portfolio (Hulme et al. 2008). For intentional introductions, understanding entrainment risk amounts to investigating the socio-political motivations for introducing new species. These are many and diverse and, through time, have ranged from agriculture and horticulture, which are ongoing (Dehnen-Schmutz 2011), to the activities of acclimatization societies (Cassey et al. 2004). In the nineteenth century, acclimatization societies were established to ‘improve’ what were considered impoverished biotas in newly colonized regions (e.g. North America, New Zealand and Australia) through the importation of plants, birds and mammals, typically from Europe, but also from other regions. Investigations of survival during transport belong in the realm either of plant propagation and animal husbandry, or maintenance of biocontrol agents (e.g. Teshler et al. 2004). While these areas may have much to inform the investigation of biological invasions and their evolutionary dynamics (e.g. Vorsino et al. 2012), we will not consider them here. Rather, our emphasis is on unintentional introductions.

Much of the understanding required to reduce the rates of unintentional introductions is concerned with improving knowledge of the pathways and vectors of invasion (Hulme 2009). Preventing an invasion is the most cost-effective way of dealing with it (Simberloff et al. 2013). These pathways and vectors are now becoming much better understood across a range of activities in different areas, and for different organisms (e.g. Drake and Lodge 2004; Tatem and Hay 2007; Huiskes et al. 2014). Changing human traffic patterns in tandem with changing climates (including their variability) is likely to change substantially the sites of entrainment risk for any given location (Tatem 2009). In consequence, although something of an invasion cycle exists, where disturbed entrainment areas tend to harbour similar suites of species (Lee and Chown 2009), forecasting which species and areas to prioritize for surveillance may be difficult because of rapidly changing environments and species distributions. Ongoing invasion of source areas (see e.g. Roy et al. 2011) may likewise mean changing risk profiles. Genomic approaches offer a powerful tool to help manage these risks.

At their most straightforward, DNA barcoding approaches can identify alien species at a given location (Darling and Blum 2007). They are now proving useful for a range of taxa. Mostly, the aim has been to characterize the diversity of an assemblage and identify alien species among its members in a given recipient area, or to verify whether given populations are from species thought to be nonindigenous (e.g. Scheffer et al. 2006; Smith and Fisher 2009; Porco et al. 2012, 2013; Fernández-Álvarez and Machordom 2013; Zhang et al. 2013a). Such approaches nonetheless are subject to the same kinds of problems as those facing DNA barcoding generally (see review by Taylor and Harris 2012). These include substantial error rates in some taxonomic groups (e.g. Hickerson et al. 2006; Meier et al. 2006; Zhang et al. 2013b) and lack of clarity about the questions being posed or rigorous independent assessment of the hypotheses being tested (Collins and Cruickshank 2013).

As DNA barcoding approaches were developed, it has now become possible to screen simultaneously samples of many species. Such metabarcoding provides relatively reliable estimates of community composition and turnover (Yoccoz et al. 2012; Yu et al. 2012; Ji et al. 2013). The approach has also been broadened to include what may be termed environmental metabarcoding or eDNA metabarcoding (Taberlet et al. 2012a; Bohmann et al. 2014). Here, rather than the organisms being sequenced directly, DNA is typically extracted from soil or water (Collins et al. 2013; Porco et al. 2013; Piaggio et al. 2014), but may also come from other sources such as the gut contents of flies (Calvignac-Spencer et al. 2013). The terms metagenomics and ecogenomics have also been applied to this approach, although typically metagenomics involves analysis of functional characteristics and assembly of whole genomes for microbes (Taberlet et al. 2012a). Considerable success has been had in detecting invasive alien species using this approach, with the method surpassing traditional survey approaches both in terms of sampling effort and sensitivity. Notable demonstrations thereof are for carp in waterways linking to the Laurentian Great Lakes (Jerde et al. 2011), the American bullfrog in France (Ficetola et al. 2008; Dejean et al. 2012) and several other aquatic species (Bohmann et al. 2014; Rees et al. 2014). Much interest exists in the application of eDNA barcoding to surveillance of ballast water in ships, a substantial source of aquatic invasions, with novel tools being developed to enable rapid screening by nonspecialists (Mahon et al. 2012).

A further area where eDNA metabarcoding is proving useful is in understanding the extent, ecology and ecosystem functioning consequences of microbial invasions (Litchman 2010; Shade et al. 2012). Microbial invasions are significant across the globe with substantial impacts on a range of ecosystems and on human and animal health (Van der Putten et al. 2007; Jones et al. 2008; Vellinga et al. 2009; Keesing et al. 2010; Cowan et al. 2011; Carroll et al. 2014). Because metagenomics has deep roots in microbiology, the approach enables sophisticated portraits to be painted of the identity of the players involved in a given system, the extent to which invasions are significant and the functional roles that key members of the community play (Litchman 2010; Cowan et al. 2011; Shade et al. 2012; Adriaennsens and Cowan 2014; Ramirez et al. 2014).

Metabarcoding and eDNA metabarcoding approaches are not without their problems, including challenges to do both with resolving taxa (see above and Taberlet et al. 2012b; Brodin et al. 2013; Pyšek et al. 2013), and with the skills, methods and databases required to ensure that these approaches deliver their full potential (Yoccoz 2012). Nonetheless, the field is developing rapidly (e.g. Liu et al. 2013; Zhang et al. 2013b; Bohmann et al. 2014), and management risks posed by differentiating among increasing numbers of species introductions and range shifts precipitated by changing climates (Walther et al. 2009), could readily be reduced using these approaches.

Although barcoding and metabarcoding approaches are increasingly being applied to the detection of invasives and have been used in several successful detections (e.g. Dejean et al. 2012; Collins et al. 2013; Egan et al. 2013; Takahara et al. 2013), they could also be used to reduce risks of introduction by understanding the composition of species in source hot spots. While doing so might at first seem impracticable, reductions in costs of the technical methods (Lemmon et al. 2012; Rocha et al. 2013), and routine surveillance of areas for incoming species (e.g. Bourlat et al. 2013), which are often the same sources for outgoing ones (Lee and Chown 2009), mean that the data may become increasingly available for species detection. The main barriers to overcome are making sure that sequence information, biological records and distributional data are available in linked and readily useable forms [although their downstream use also faces a variety of challenges (Amano and Sutherland 2013)], achieving interoperability among the various approaches and information databases, and estimating the extent of errors of omission and commission for various taxa (Collins and Cruickshank 2013). While these barriers are high, they are being reduced by increasing discussions among major data providers and ongoing work to improve interoperability. Although this provider approach is important, good practice by investigators (such as coherence in data submitted to major providers) is probably the most significant way to reduce barriers. DNA barcoding approaches may be especially helpful where changing climates encourage change in human agricultural and business practices, creating rapidly changing invasion scenarios.

## Identifying source populations

**Figure 2:**
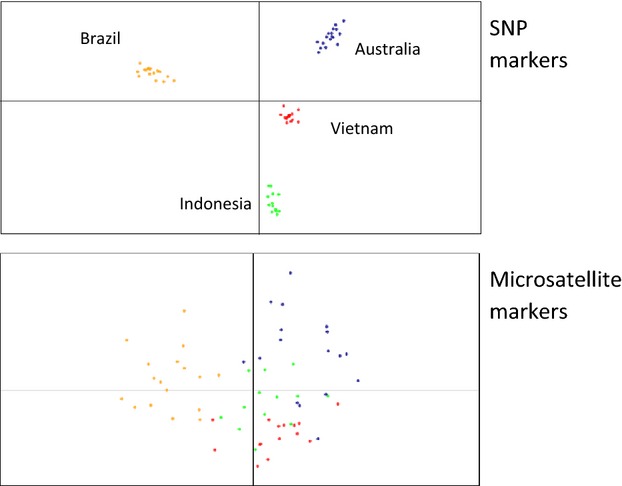
Comparison of population structure of *Aedes aegypti* mosquitoes as determined from microsatellite markers versus SNP markers. Based on a discriminant analysis, the SNP markers provide a far higher level of resolution of populations including the invaded (non-Asian) range of this species (from Rasic et al. 2014).

Although genetic markers have been used for some time and are now used almost routinely for identifying source populations of species, genomic markers promise a much higher level of resolution and they are being increasingly applied. A recent example is provided by the dengue vector mosquito, *Aedes aegypti*, whose source populations have traditionally been identified using mtDNA markers coupled with microsatellites (Endersby et al. 2011; Brown et al. 2014). These provide some indication of population sources and are currently used in countries such as Australia to locate source populations. However, a much higher level of resolution for identifying source populations can be obtained from SNP nuclear markers that can now be scored in the thousands; this was demonstrated in the extent to which geographic samples of *Ae. aegypti* could be completely resolved based on 18 000 SNP markers compared to 10–15 other markers (Rasic et al. 2014) (Fig.2).

SNP markers are now being developed for other invasive species including invasive plants (De Kort et al. 2014) and mammals (White et al. 2013) as well as insects. These markers promise to revolutionize risk assessment and quarantine control because they can reveal both contemporary and historical population processes. The potential of this approach in detecting and understanding past patterns based on whole-genome sequencing is evident from recent human studies on invasions and contractions of different cultural groups that provide new insights into patterns of relatedness and the spread of cultural practices (Sikora et al. 2014).

Understanding source populations and spread dynamics may benefit from using nuclear and extranuclear genomes. For instance, in the Russian wheat aphid, Zhang et al. (2014) tested patterns of invasion by characterizing more than 500 clones for genetic markers in nuclear DNA, mtDNA and endosymbionts. They were able to show that Turkey and Syria were the most likely sources of invasion to Kenya and South Africa respectively, as well as establishing patterns of invasion into the New World. Having the different sources of genomic information was essential to this study as the recent introduction limited the genetic resolution of the aphid markers used. Combining nuclear DNA and organellar markers has traditionally improved the understanding of invasive processes, and it is becoming clear that including vertically transmitted endosymbiont or parasitic organisms can provide new insight into the origins of invasive species as well as the diseases they carry (Gupta et al. 2014; Mobegi et al. 2014). The addition of mtDNA provides information on introgression patterns between diverged populations or subspecies because mtDNA genomes track maternal lineages (McCormick et al. 2014).

In the future, genomic information will likely prove useful in identifying source risk and predicted outcomes of invasions when they are detected. For instance, in ragweed, it appears, based on microsatellite and chloroplast markers, that invasive populations have developed in the native range of this species associated with human habitation, and these populations are then likely to form the basis of invasive populations into other areas (Martin et al. 2014). Predictions are that invasions will grow in number and be increasingly difficult to distinguish from range shifts (Walther et al. 2009). Genomic techniques that can facilitate rapid identification of source populations will prove especially useful for addressing this problem (for an application in identifying the basis of range expansion, see Krehenwinkel and Tautz 2013).

## Colonization and establishment: genetic diversity

Multiple founders can facilitate population establishment in two ways. First, propagule pressure mitigates the impact of demographic stochasticity; second, increased genetic and phenotypic variation improves the chances of successful colonization (Colautti et al. 2006; Simberloff 2009; Rius and Darling 2014). A recent meta-analysis, which controlled for the effects of population size, found strong evidence that higher levels of genotypic and phenotypic diversity in founder groups increased establishment success (Forsman 2014). Genetic variation can improve the likelihood of establishment for many reasons (Zhu et al. 2000; Willi et al. 2006; Gamfeldt and Källström 2007; Dlugosch and Parker 2008; Prentis et al. 2008; Thompson et al. 2012; Berthouly-Salazar et al. 2013), and evidence is mounting that a large proportion of invasive species have experienced multiple introductions (e.g. Bossdorf et al. 2005; Roman and Darling 2007; Thompson et al. 2012; Rius and Darling 2014).

The role of genetic variation in successful invasions has proven contentious though, with authors often taking opposing views depending on the nature of the system that they study (Rius and Darling 2014). Indeed, many species have spread successfully with low genetic diversity (Myburgh et al. 2007; Darling et al. 2008a; Richards et al. 2012). The success of invaders with low genetic variability may depend on the nature of the environment into which they invade; weedy species moving into agricultural landscapes may require less genetic variability because they enter a relatively homogeneous landscape, whereas for species invading natural environments, a high level of variability may be required (Moran and Alexander 2014).

Genomic approaches provide a means to help resolve the apparent paradox. For example, success with limited genetic diversity may be due to the expression of phenotypic plasticity, aided by epigenetic responses to the environment (Richards et al. 2012) that can be identified with genomic approaches. Epigenetic mechanisms may be particularly important in these situations because they generate heritable changes in response to environmental cues, although their ecological significance remains unclear (Bossdorf et al. 2008). Alternatively, functional traits may remain variable even when genetic variation is low, as the loss of rare alleles will not greatly affect the quantitative trait distribution, and nonadditive genetic variance may either aid in fitness directly, or be converted to additive variance due to allele frequency shifts (Dlugosch and Parker 2008; Dawson et al. 2012; Le Roux et al. 2013). Genomic tools provide a way of identifying variation in functional traits (see next section). They also provide new means to understand the nature of introductions of invasive species. As mentioned in the previous section, genomic markers provide a much higher level of resolution when identifying source populations, and they can therefore clearly indicate the number of introductions from different environmental regions involved in invasions.

## Colonization and establishment: adaptive loci

Identifying risk factors associated with establishment would be assisted by more information on the genetic basis of adaptive changes in colonizing species. If these changes were known and repeatable, it might help predict when colonizations occur. One of the best examples is the evolutionary changes involved in the repeated independent colonization of freshwater environments by sticklebacks (summary in Bell and Aguirre 2013). These colonization events have been associated with repeatable changes in lateral plate morphology across populations involving the ectodysplasin (EDA) locus. Many loci in different genomic regions also appear to be involved, but nevertheless there appear to be changes across the same set of genomic regions in different, isolated freshwater populations of sticklebacks following invasion (Jones et al. 2012). The stickleback situation may be unusual because there has been a repeated process of invasions that have been occurring over 10 million years (Bell and Aguirre 2013), but more cases need to be examined. Because invasive species often occupy similar habitats in the introduced range to those in the native range (see Guisan et al. 2014 for a recent overview and perspective), there is an opportunity to determine whether the same genomic regions contribute to adaptations in the native and introduced ranges across many invasive species.

While there is abundant evidence for changes in neutral markers following invasion, characterization of adaptive molecular differences, such as those established in stickleback populations, remains relatively scarce. Transcriptome comparisons of plants in common garden experiments suggest that invasive populations of ragweed show altered patterns of gene expression that are distinct from most populations from the native range, and genes showing altered patterns of expression appear to be linked to stress and biotic responses (Hodgins et al. 2013). A similar pattern has since been identified in Canada thistle (Guggisberg et al. 2013), but whether changes to expression during invasion primarily involve certain functional groups, as indicated by these studies, will require more examples across a broader array of organisms.

One of the challenges in finding the genetic basis of adaptive change is that causal connections between genetic changes and traits can be difficult to identify. Often researchers are faced with the problem of locating small but crucial changes within a mass of genomic data. However, the implementation of population genomic analysis of SNP variation across the genome has enabled the identification of putatively selected loci (or those linked to the causal loci). This approach has already proven useful in several cases. Pyrenean rocket (*Sisymbrium austriacum* subsp. *chrysanthum*) is a small colonizing herb of rocky soils that has recently invaded Belgium and the Netherlands, and Vandepitte et al. (2014) used a RAD-Seq approach to identify outlier loci in comparisons between invaded and native populations. Multiple native and invaded populations were investigated including those represented by herbarium specimens covering *ca*. 100 years in the invasive range. Several outlier loci that could underlie adaptive differences were discovered, and the sampling enabled a comparison of outliers across space as well as time. Some of the SNPs were located in genes known to affect flowering time in the closely related *Arabidopsis thaliana*. These findings point to likely adaptive shifts rather than changes associated with demographic processes. This study demonstrates the utility of phenotype–genotype approaches for identifying candidate adaptive loci in invasive species coupled with functional information from model species. As the genomes of invasive species become better defined and genetic tools are developed, it should be increasingly possible to also pursue bottom-up approaches in a range of invasive species particularly because these can often be relatively easily grown under controlled conditions.

## Colonization and establishment: hybridization and polyploidy

Hybridization can act as a stimulus to invasion (Ellstrand and Schierenbeck 2000; Schierenbeck and Ellstrand 2009) and occurs on a continuum from the combining of largely reproductively isolated genomes to the mixing of distinct populations within a species. Hybridization offers many potential advantages during invasion. It may increase adaptive potential through the injection of genetic variation and the formation of beneficial gene combinations (Anderson 1948). Hybridization is known to result in transgressive segregation, where phenotypic variation in the hybrids exceeds that of the parents (Rieseberg et al. 2003). In sunflowers and Louisiana irises, there are particularly compelling cases for hybridization facilitating the colonization of novel habitats (Rieseberg et al. 2003, 2007; Arnold et al. 2012).

Over the short term, heterosis (improved performance of hybrid offspring) could be important for overcoming demographic stochasticity associated with initial establishment (Drake 2006; Keller and Taylor 2010; Rius and Darling 2014). Although heterosis is transitory in most cases (Hochholdinger and Hoecker 2007), fixed heterozygosity can be maintained across generations in allopolyploids (assuming disomic inheritance occurs) and asexually reproducing species (Ellstrand and Schierenbeck 2000; Rieseberg et al. 2007). In addition, if dominance is the primary cause of heterosis, the advantage of hybridization may continue in later generations and facilitate the purging of genetic load (Burke and Arnold 2001). Alternative mildly deleterious alleles can become fixed within diverging populations due to the effects of genetic drift. Hybridization between these lineages could produce descendants with an intrinsic fitness advantage over their parents that is maintained if the combined effects of recombination and natural selection erode the frequency of these deleterious alleles (Ellstrand and Schierenbeck 2000; Burke and Arnold 2001).

Genomic tools can help in identifying the consequences of these processes because they can be followed across different parts of the genome. This applies particularly to plants where invasive taxa often arise as hybrids (Moody and Les 2007; Ainouche et al. 2009; Schierenbeck and Ellstrand 2009). One famous case is of the damaging riparian invasive *Tamarix* species in the USA. The main invasive lineage arose from hybridization between two introduced species, *T. chinensis* and *T. ramosissima*, which have largely separate ranges with some overlap in Asia, but do not appear to hybridize there (Gaskin and Schaal 2002). Genome-wide neutral markers revealed that hybrids dominate the invasive range, and the level of introgression is strongly correlated with latitude (Gaskin and Kazmer 2009). A corresponding latitudinal cline in cold hardiness suggests that hybridization may have contributed to the rapid adaptation of *Tamarix* to climatic extremes in North America (Friedman et al. 2008). In reed canarygrass, multiple introductions into North America of genetic material from different European regions have mitigated the impact of genetic bottlenecks (Lavergne and Molofsky 2007). This species has higher genetic diversity and heritable phenotypic variation in its invasive range relative to its native range. Comparisons of neutral differentiation (*F*_ST_) to differentiation in quantitative traits among populations (*Q*_ST_) provide evidence for rapid selection of genotypes with greater vegetative colonization ability and phenotypic plasticity in the introduced range.

Hybridization is expected to be important from the perspective of invasiveness under climate change for two reasons. First, evidence is growing that climate change is increasing rates of hybridization, as species that were previously geographically isolated come into contact with each other (Garroway et al. 2010; Muhlfeld et al. 2014). Second, evidence from genomic studies is revealing that genes associated with climate change adaptation can be of hybrid origin (Becker et al. 2013; De La Torre et al. 2014). Thus, when hybrids are formed, they may contribute to rapid adaptation under climate change. Although most information on hybridization comes from plants, hybridization is also likely to play an important role in the adaptation of animal lineages to climate change. Genomic comparisons have indicated introgression of invasive genes across lineage boundaries affected by climate in birds (Taylor et al. 2014) and stick insects (Nosil et al. 2012). Introgressed genotypes can be favoured under changing climatic conditions as evident in hybridization events in swallowtail butterflies (Scriber 2011) and in *Anopheles* mosquitoes (Besansky et al. 2003). Genome scans across multiple related species along climate gradients are likely to provide many other examples of candidate genomic regions associated with climate adaptation and exchanged through hybridization and introgression.

Polyploidy has occurred frequently in the evolutionary history of angiosperms, often associated with hybridization events (Wood et al. 2009). There are several examples of recently derived invasive polyploids (Ainouche et al. 2009; Schierenbeck and Ellstrand 2009; te Beest et al. 2012), and polyploidy is over-represented among alien plant taxa (e.g. Pandit et al. 2011). Polyploidy is associated with several advantages (reviewed in Otto and Whitton 2000; Comai 2005; Otto 2007; te Beest et al. 2012) that could aid in invasion, including pre-adapting species to the environmental conditions in the new range (e.g. Henery et al. 2010), masking deleterious alleles (Otto and Whitton 2000; Otto 2007), restoring fertility through chromosome doubling (e.g. Ainouche et al. 2009) or through changes to the reproductive system to that contribute to colonization ability (e.g. Robertson et al. 2011). In addition, allopolyploids benefit from fixed heterozygosity due to the combining of divergent parental genomes. This feature may have contributed to the evolutionary success of polyploids over diploids in the Arctic enabling them to survive dramatic shifts in climate (Brochmann et al. 2004).

One of the best examples of polyploidy contributing to invasion success is tetraploid spotted knapweed, *Centaurea stoebe*. Although both diploids and tetraploids were introduced into the USA from Europe, invasive populations are dominated by tetraploids (Treier et al. 2009). Tetraploids are pre-adapted to a wider range of climates compared to diploids, and following introduction, they may have further adapted to their introduced environment (Treier et al. 2009; Henery et al. 2010). A classic example of hybridization and polyploidy coinciding with invasion is *Spartina anglica*. This species is a recently formed allopolyploid (12×) that is highly invasive compared to its parental species, is able to colonize a broader range of habitats and exhibits substantial phenotypic plasticity (Thompson 1991; Ainouche et al. 2009), despite a strong genetic bottleneck that occurred during its formation (Baumel et al. 2001). Neo-allopolyploids often undergo extensive alterations to their genome and transcriptome, and genomic studies of *S. anglica* are dissecting the genetic, epigenetic and expression changes following hybridization and genome duplication (Salmon et al. 2005; Ainouche et al. 2009; Chelaifa et al. 2010). However, the mechanism by which *S. anglica* has become invasive remains elusive, and it is not known whether the success of this species reflects fixed heterosis and restored fertility of the hybrid following genome duplication, or phenotypic novelty due to epigenetic and transcriptome remodelling.

Whole-genome and reduced-representation genomic approaches are invaluable for detecting hybridization, and more powerful than any other method to quantify the extent of introgression of genomic material and (potentially) functional genes involved in environmental tolerance and invasiveness. They have been used to estimate rates and genomic extent of hybridization (Larson et al. 2013; Parchman et al. 2013) and identify the potential for genetic swamping, or hybrid incompatibilities that may be deleterious to native species (Fitzpatrick et al. 2010). Applying genetic and genomic methods to recent polyploids poses extra challenges due to the presence of multiple genome and gene copies; this can complicate short-read alignment and *de novo* assembly of genomes, but can also be useful in establishing patterns of relatedness among species and populations (Griffin et al. 2011). Reference genomes are especially valuable tools in these cases and are helping to reveal within-species diversity and hybrid origins of polyploids (e.g. Evans et al. 2014; Marcussen et al. 2014), and reduced-representation library methods can now be applied even without a reference genome (Lu et al. 2013). Genomic studies of several plant groups are pointing to porous species barriers across thousands of years where ongoing hybridization and gene flow between species is present despite species maintaining their morphological integrity (e.g. De La Torre et al. 2014; Griffin and Hoffmann 2014). Such a dynamic process of hybridization and polyploidy may also occur among different chromosomal races within a species as in the case of buffel grass, which is weedy and invasive in some areas of its current range and consists of different polyploids that vary in their environmental distributions and invasiveness (Kharrat-Souissi et al. 2014).

## Colonization and establishment: phenotypic plasticity

Although the link between phenotypic plasticity and distribution has not been comprehensively supported (Dawson et al. 2012), phenotypic plasticity clearly contributes to environmental tolerance in various ways (e.g. Ghalambor et al. 2007; Des Marais et al. 2013; Schilthuizen and Kellermann 2014). Moreover, at least under conditions of resource abundance, it appears that invasive species do show greater plasticity than their indigenous counterparts (Davidson et al. 2011), although the way in which such comparisons should be made has been the subject of much recent discussion (Van Kleunen et al. 2010; Leffler et al. 2014).

Phenotypic plasticity plays a significant role in the responses of both plants and animals to environmental change (De Witt and Scheiner 2004; Ellers and Stuefer 2010; Nicotra et al. 2010). The extent to which plasticity promotes longer-term adaptation may vary, however, ranging from cases where it promotes adaptation through to those where it might inhibit adaptation. The outcome depends on a range of circumstances, including the extent and form of environmental predictability, the extent of dispersal and the genetic variance in new environments (Chown and Terblanche 2007; Ghalambor et al. 2007; Scoville and Pfrender 2010; Chevin et al. 2013). Nonetheless, currently, it appears that phenotypic plasticity can play a major role in species responses to climate change, such as in changing the timing of breeding in birds (Gienapp et al. 2008) and responses of plants along climate gradients (Matesanz and Valladares 2014; McLean et al. 2014). Some evidence also exists suggesting that invasive species have advantages over indigenous ones as a consequence either of the extent or form of their plasticity (Stachowicz et al. 2002; Chown et al. 2007; Kleynhans et al. 2014). Given that at least some evidence supports the idea that phenotypic plasticity may enhance the success of nonindigenous species as climates change, an understanding of the genomic basis of plasticity may improve forecasts of where such advantage should be expected and what form it should take.

The genomic and transcriptomic basis of variation in phenotypic plasticity is still poorly understood. Several gene expression studies have now been undertaken across related species or populations exposed to different environmental conditions to assess whether these might provide clues about the mechanistic basis of variability in plastic responses (Smith et al. 2013; Dunning et al. 2014; Meier et al. 2014). A few of them have considered populations from native and invaded parts of their range to assess whether there are differences in plastic responses to the different environmental conditions encountered in invasive situations. For instance, in sticklebacks, the invasion of freshwater environments by marine populations has involved an increase in expression plasticity, including some genes that are thought to be involved in adaptation to these new habitats (Morris et al. 2014), while in *D. melanogaster* populations from warm environments, there was an enrichment of down-regulated genes when flies were raised in conditions that were not commonly encountered in their native range (Levine et al. 2011).

A challenge is to link these expression changes to adaptive variation in plasticity; population differences in gene expression under different conditions may indicate adaptive changes in plasticity, but expression changes have not yet been clearly linked mechanistically to such changes. One problem in making such connections is that there is a level of complexity in organism's transcriptome that largely remains to be explored even in model organisms. For instance, in *D. melanogaster,* the transcriptome can be modified qualitatively in a variety of ways, such as through alternative splicing and expression of alternative isoforms, that may ultimately have more impact on plastic responses than quantitative changes in gene expression. These sources of variation for climate adaptation are only just starting to be explored (Telonis-Scott et al. 2013).

Another challenge is to consider plastic changes across generations as well as within them. Epigenetic modifications such as DNA methylation and histone modifications can be induced by environment and influence gene expression and transposable element activity, with direct effects on the phenotype (Glastad et al. 2011; Herrera et al. 2012; Zhang et al. 2013c). In plants, methylation can also be inherited through multiple generations, thus partly acting like heritable genetic variation. There are several examples of invasive species where epigenetic variation far exceeds standing genetic variation (Richards et al. 2012; Liebl et al. 2013). Genomics is integral to the field of ecological epigenetics. Whole-genome bisulphite sequencing can reconstruct entire methylation profiles (Schmitz et al. 2013; Wang et al. 2013) while reduced-representation genomic methods and genetic methods such as MS-AFLP can enable quantification of the extent of epigenetic compared to genetic variation within and among populations (Pérez et al. 2006). The hypothesis that epigenetic variation is important to invasion in some cases is intriguing, and a handful of studies exist directly linking epigenotype to phenotypic means, plasticities and environmental tolerance (Herrera et al. 2012; Zhang et al. 2013c), but in general, few data have been collected that examine adaptive and heritable epigenetic effects (Furrow and Feldman 2014).

## Spread

Understanding the spread/expansion phase of an invasion has similarities with species range projections in climate change biology (Caplat et al. 2013). To expand its range into new environments, a species requires appropriate dispersal traits and tolerance of a range of environments or capacity to experience the new environment as consistently receptive. Dispersal traits are a key characteristic of invasive species, and dispersal occurs through direct movement (in the case of animals) or indirect movement, by seed or vegetative dispersal (in the case of plants), dispersal through soil or water (e.g. the plant pathogen *Phytophthora cinnamomi*) or human-mediated dispersal (e.g. weedy species along roadsides, cane toads in transport vehicles and vertebrates on ships).

Genomic studies on populations can be used to test ecological hypotheses about dispersal patterns in invasive species once these have become established in a new area and started to spread, including the widely documented lag phase before populations disperse widely (Richardson et al. 2011). For instance, Rohfritsch et al. (2013) used genomic data to map the spread of an invasive oyster across Europe following its introduction from Asia. This approach is particularly powerful when combined with ecological models that predict the extent to which populations are expected to occupy new space across time or maintain refugia under a changing climate, both of which should be reflected in patterns of genetic variation (Fordham et al. 2014). For example, genomic approaches have been used to understand patterns of spread in Australian *Acacia* species that have subsequently invaded southern Africa. These include estimating the impacts of human-assisted dispersal in the native range prior to introduction (Le Roux et al. 2013) and better forecasting of the effects of climate change by accounting for genotypic variation (see Millar et al. 2011; Thompson et al. 2011).

Population genomic data can also be used to understand the impact of spreading invaders through hybridization and introgression. As discussed above, invaders spreading into non-native habitat, particularly under climate change, are expected to hybridize with local species where reproductive isolation is incomplete. This can lead to the incursion of genes from the invaders into native species. As a consequence, local species can become genetically ‘polluted’, resulting in the loss of species integrity. Genomic markers provide a way of detecting such pollution (e.g. Yamazaki et al. 2005; Sampson and Byrne 2008; Millar et al. 2012).

Mechanisms of dispersal can evolve, as in the case of leg length in the cane toad (Phillips et al. 2006), and seed dispersal in *Abronia umbellata* (Darling et al. 2008b). In these cases, genomic studies on quantitative traits can provide information on the genes and pathways involved. Dispersal evolution may involve traits directly involved in movement, such as cane toad leg length, or more subtle changes that nevertheless enhance dispersal potential, such as altered timing of reproduction that increases seed dispersal. Genomic data can identify the nature of these evolved changes in mechanisms that alter the potential of populations to spread. For instance, QTLs have been isolated that control the formation of rhizomes in perennial wild rye that represents an important trait facilitating colonization in weedy ryegrass species (Yun et al. 2014). Understanding the genomic basis of traits involved in non-human-mediated dispersal may aid in predicting the invasive potential of suspect species before introduction or at the establishment stage (Pérez et al. 2006), as well as projecting their future spread in cases where dispersal traits can evolve.

## Management implications

The broad consensus in the literature is that climate change is likely to interact synergistically with biological invasions, leading to increasing economic and biodiversity impact (Walther et al. 2009; Scholes et al. 2014). Much evidence exists for recent changes in pest and disease distributions (Bebber et al. 2013), although substantial variation exists among taxa and geographic regions in the extent to which an overall increase in the burden of invasives under climate change might be expected (Bellard et al. 2013). A complicating factor is that human activity in the form of trade, transport and adaptation to climate change is likely to increase too. This will complicate decisions about range shifts relative to anthropogenic introductions and thus potentially confounding management decisions that are required to limit the overall burden of invasive species impacts. Even in relatively remote areas, these problems are playing out (Chown et al. 2012). Overall, the prospects are for increased impacts of invasion as climate change proceeds (Scholes et al. 2014).

From a management perspective, prevention, usually involving some form of risk assessment, is clearly the most cost-effective solution to invasions (Simberloff et al. 2013). Therefore, considerable focus should be on prevention including the identification of the source and receiving areas for introductions (such as ports). Internationally, risk assessment is accepted as an essential policy instrument for managing biological risk. Risk is quantified by examining the invasiveness, impacts and potential distribution of species, areas which are all being improved by the adoption of genomic approaches. Thereafter (i.e. in a postborder context), substantial focus on interventions across the stages of invasion is critical for management success both in relation to typical population processes (such as those involved in emergence from lag phases) (Kueffer et al. 2013; Ricciardi et al. 2013; Simberloff et al. 2013) and to changes in the environment associated with climate change and human adaptation to such change. Likewise, understanding when invasion success may be driven by factors other than climate change, such as local adaptation to climates, evolution of increased competitive ability and enemy release (Blossey and Notzold 1995; Chun et al. 2010; Van Kleunen et al. 2010; Hill et al. 2013), is also important for managing invasions. Prioritization of management actions to species of greatest risk (Randall et al. 2008; Byrne et al. 2011; Forsyth et al. 2012) is critical given the requirement for effective outcomes with investment of limited resources. As hybridization has been demonstrated to facilitate invasiveness, assessment of genetic risk is an important management tool (Byrne et al. 2011), particularly in the context of managed relocation as a climate change adaptation strategy (Schwartz et al. 2012).

Throughout this review, we have demonstrated how genomics approaches can improve understanding, and in many cases forecasting, at each stage of invasion (Fig.1). These include better understanding of source areas and the identity of introduced species. In this sense, genomic approaches are typically improvements on genetic tools that have been available for sometime; they allow source populations to be identified more accurately, provide a clearer picture regarding multiple interactions, including the role of hybridization, and lead to better estimates of population size, gene flow and other processes. NGS techniques can provide ways of detecting invasive organisms that have previously not been available, and identifications become more accurate as additional sequence information is added. Microsatellite and mtDNA markers have been widely used to understand the population dynamics of agricultural pests and disease vectors in the past and thereby assisting in programs aimed at controlling pests and/or limiting their impact. However, the new genomic tools provide an unprecedented view of past and present population processes (e.g. Brown et al. 2014; Rasic et al. 2014). With invasion frequency increasing under climate change, these are all important tools in improving an understanding of invasion processes.

Where control of invasive species is being considered, genomic approaches can help in defining units for eradication and risks of reinvasion once eradication is achieved (Fig.1). At present, markers such as microsatellites are often used for defining movement patterns among populations of invasive pest mammals (e.g. Hampton et al. 2004; Abdelkrim et al. 2005; Berry et al. 2012; Adams et al. 2014). With the use of genomics approaches, managers should become much more confident of likely movement patterns and reinvasion potential as local movement patterns can be tracked much more accurately than in the past.

However, genomics tools promise much more than ways of simply improving detection and an understanding of spread. High-density markers provide a way of tracking changes in different parts of the genome, essential for understanding hybridization and introgression as well as adaptation under climate change including the application of quantitative genomic approaches to identify regions with candidate genes (Fig.1). This can be used to understand the roles of various evolutionary processes at key steps of the invasion pathway, and the ways in which climate change will interact with the latter. Key areas include investigation of evolutionary change underpinning increased competitive ability and enemy release, especially in a biological control context. Genomics methods can also help redirect resources by improving understanding of the burden of invasion, an especially important role as that burden increases (e.g. Pfenninger et al. 2014). They can assist with rehabilitation in the case where substantial invasive impacts result in widespread ecosystem changes, by understanding patterns of resistance and identifying resistant genotypes that can persist into the future. For example, where trees are affected by introduced pathogens, there is the possibility of identifying tree genotypes that survive outbreaks (Stukely and Crane 1994; McKinney et al. 2014). As information becomes available on adaptive changes in traits controlling the spread and climate change adaptation, it should also be possible to refine models about the non-native range of an invasive species. For instance, Kearney et al. (2009) modelled the expected range of *Aedes aegypti* in northern Australia based on anticipated evolutionary changes in egg desiccation tolerance under climate change and showed the potential threat posed by this species around a population centre.

In summary, genomic tools are proving to be extremely useful for managing invasive species. While they may be seen as costly for less well-resourced nations, several studies have demonstrated that they are more cost-effective than traditional surveillance tools and other methods. Moreover, technology is now advancing to the stage where users do not need to be familiar with its intricacies (in much the same way, most mobile phone users could not build one), and substantial programmes of technology assistance are in place through various international agencies (such as the Consultative Group on International Agricultural Research, www.cgiar.org). In consequence, the myriad ways genomic tools can be utilized will enable society to contain the economic cost of biological invasions accentuated by climate change, and prevent a world dominated by weedy species.
